# Un legado de 80 años y pasos firmes hacia el futuro

**DOI:** 10.31053/1853.0605.v80.n4.43883

**Published:** 2023-12-26

**Authors:** Andrea Martínez, Natalia Traversaro, Viviana Dugatto

**Affiliations:** 1 Editora Técnica Revista de la Faculta de Ciencias Médicas de Córdoba; 2 Universidad Nacional de Córdoba. Facultad de Ciencias Médicas. Biblioteca “Prof. Dr. J. M. Allende”; 3 Universidad Nacional de Córdoba. Facultad de Ciencias Médicas. Secretaría de Ciencia y Tecnología

Durante ocho décadas, la Revista de la Facultad de Ciencias Médicas de Córdoba ha sido un pilar fundamental en la difusión del conocimiento científico en el ámbito académico de la Facultad de Ciencias Médicas de la Universidad Nacional de Córdoba.

La transformación digital desde el año 2005 se ha traducido en una evolución desde aquella primera publicación impresa en 1943 hacia un formato que abarcó tanto el papel como electrónico, convirtiéndose éste último en el definitivo, a partir de la incorporación de la página web oficial. La simultaneidad entre estos dos formatos inició en el año 2005. En principio, la página web estaba alojada dentro del sitio de la Biblioteca de la Facultad de Ciencias Médicas "Prof. Dr. J. M. Allende": www.fcm.unc.edu.ar/biblio/index.html, donde se podía acceder al contenido de la revista en texto completo en formato pdf. Los envíos de artículos, correcciones y revisiones se hacían a través de correo electrónico.

A partir del año 2009, la página tuvo un dominio independiente de la biblioteca: http://www.revista.fcm.unc.edu.ar. La nueva página web incluyó información más completa sobre el equipo editorial, eISSN activado e incorporó publicidades de importantes instituciones de Córdoba. Su identidad visual respondía a los requerimientos de calidad de los organismos evaluadores ( Figura: 1 y 2).

**Figura Nº1:**
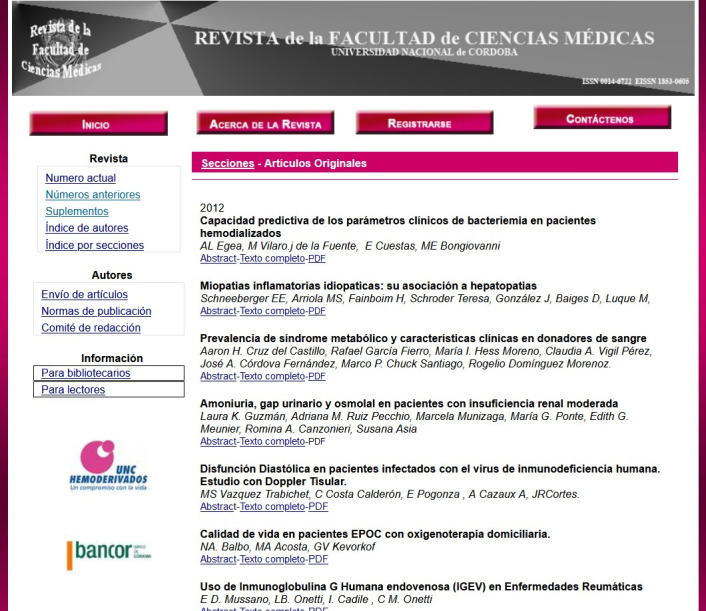
Interfaz de la Revista de la Facultad de Ciencias Médicas. Aproximadamente desde el año 2009 al 2015

**Figura N°2:**
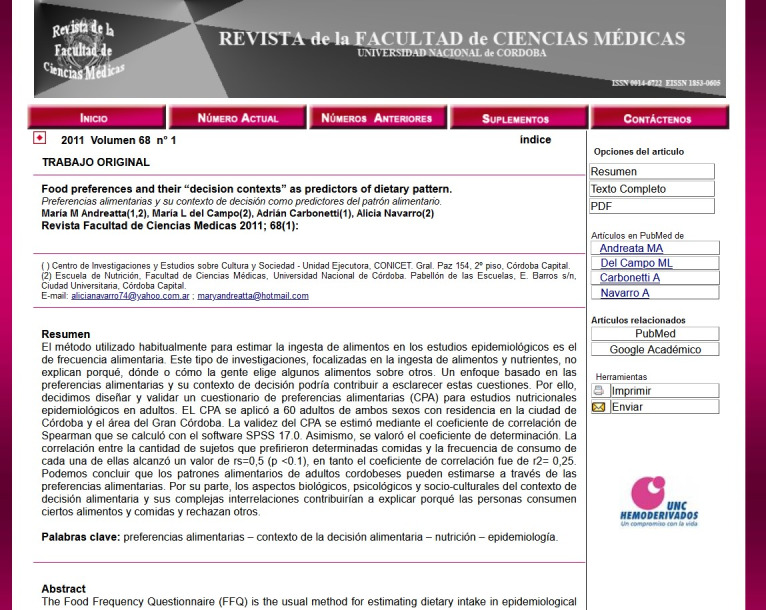
Vista de un artículo publicado con la interfaz utilizada desde el año 2009 al 2015.

La adopción del formato digital no solo representó un cambio notable en la evolución de la revista. A partir de 2016, la implementación del sistema Open Journal System (OJS) marcó un hito crucial al modificar, agilizar y facilitar el proceso editorial.

La plataforma OJS permite realizar el proceso editorial completo dentro del sistema y a la vez ofrece una interfaz que no solo muestra el contenido de la revista, además permite la realización de búsquedas y organiza la información de manera lógica y amigable, permite accesibilidad desde cualquier parte del mundo, la capacidad de realizar actualizaciones, interactividad, incorporación de métricas, etc. http://www.revista.fcm.unc.edu.ar/ (Figuras 3 y 4).

**Figura N°3.**
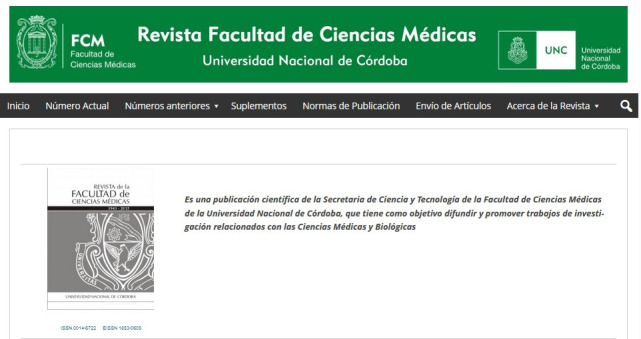
Interfaz utilizada desde el año 2015-2016.

**Figura N°4.**
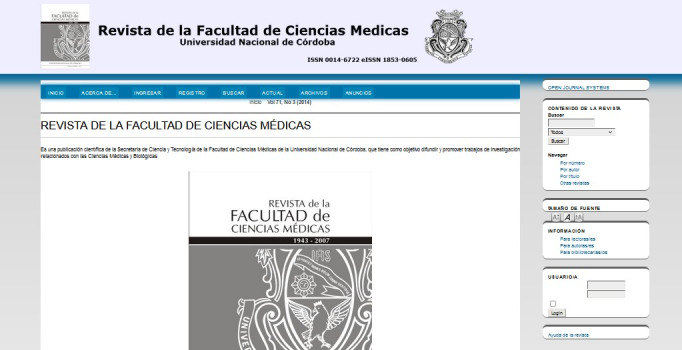
Interfaz de la RFCM con la plataforma OJS (beta) utilizada desde el año 2015-2016.

Además, la RFCM se incorporó al Portal de Revistas de la Universidad Nacional de Córdoba, lo que permitió abrir las puertas a una audiencia más global. Este paso trascendental no solo robusteció su presencia, sino que también reafirmó su compromiso con la accesibilidad y difusión del conocimiento. Este avance crucial potenció la visibilidad y el acceso a la revista. De esta manera, se consolidó como una publicación de acceso abierto que, en concordancia con las políticas institucionales de la universidad anfitriona, no impone tarifas ni por su publicación ni por el acceso a su contenido. Respaldada por esta institución, ha adoptado estándares que la han convertido en una fuente de información científica confiable y altamente respetada.

En el año 2016 también, la revista fue incluida en el Núcleo Básico de Revistas Científicas Argentinas (NBRA) - CAICYT, lo que posibilitó reafirmar su relevancia en el ámbito científico nacional e internacional.

Al ser una plataforma colaborativa y en constante crecimiento, el OJS incorpora actualizaciones que se ven reflejadas tanto en el backend ^[1]^ como el frontend ^[2]^ . Estas mejoras permiten la incorporación de funcionalidades antes inexistentes y también modifica la parte visual de la revista ( Figura: 5 y 6).

**Figura N° 5.**
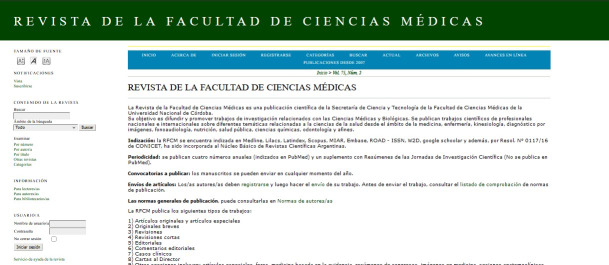
Interfaz de la RFCM con la plataforma OJS a partir del año 2016.

**Figura N° 6.**
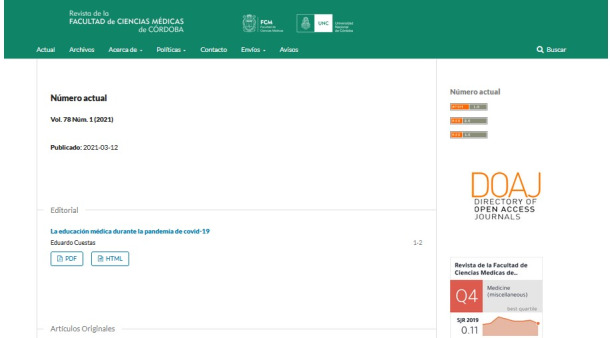
Interfaz de la RFCM con la plataforma OJS a partir del año 2021

Con el objetivo de posibilitar la circulación y acceso abierto del conocimiento y cumplir con los requerimientos de calidad a nivel internacional, la RFCM ha realizado constantes y continuas innovaciones: obtuvo el ISSN electrónico, el DOI (Digital Object Identifier) ^[3]^ ; incorporó el uso de lenguajes de marcación como el HTML y el XML, que permiten describir y estructurar la literatura científica que se publica en línea a los fines de poder migrar estos datos a otras bases de difusión.

En 2022, fue evaluada por National Center for Biotechnology Information de la National Library of Medicine y obtuvo el acceso a uno de los repositorios digitales más importantes en el área de las ciencias de la salud: "Pubmed Central". Este repositorio de acceso abierto pertenece a la Biblioteca Nacional de EEUU y Centro Nacional para Información de Biotecnología (NCBI), que publica 9,5 millones de artículos en texto completo de revistas biomédicas y ciencias de la vida. Esto permitió que además los artículos se editen en formato epub. Constantemente. se realizan adecuaciones para que la revista permanezca indizada en las bases de datos en las que ya fue evaluada como: Doaj (Directory of Open Access Journals), PubMed Central, PubMed/Medline, Latindex Directorio y Catálogo 2.0, MIAR (Matriz de Información para el Análisis de Revistas), el Directorio de Recursos Académicos de Acceso Abierto (ROAD de ISSN), Google Schoolar**,**entre otros.

Esta trayectoria de la RFCM demuestra la premisa de compromiso con la excelencia científica, la mejora continua y el prestigio de la Facultad de Ciencias Médicas de Córdoba.

A partir de 2023 se estableció un nuevo cambio de imagen significativo, motivado por diversas razones, como la adaptación a nuevos tiempos, la modernización estética, la alineación con los valores y objetivos actuales de la facultad y los requerimientos internacionales para las revistas científicas (Figura7).

**Figura N° 7:**
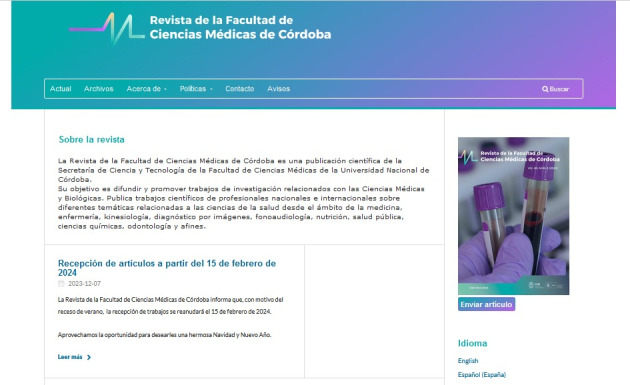
Apariencia actual (desde abril 2023) de la Revista de la Facultad de Ciencias Médicas de Córdoba.

El cambio de imagen incluye un nuevo logo, pantone de colores nuevos.

Para sumar este cambio se modificaron las normas editoriales con plantillas que facilitan a los autores el envío de artículos, tutoriales que guían en los procesos de trabajos de los autores, revisores y editores.

Los cambios y mejoras propuestos son una estrategia que busca ajustar la percepción y la representación visual de la revista y brindar herramientas que faciliten el proceso editorial a autores/as, editores/as, revisores/as. Esto posibilita adaptarse a los nuevos tiempos y tendencias actuales, con el fin de mantenerse relevante, atractiva y de ágil manejo para sus usuarios.

En conclusión, los 80 años de la Revista de la Facultad de Ciencias Médicas de Córdoba representan un legado de compromiso con la difusión del conocimiento, la búsqueda de la excelencia científica y la adaptación a los desafíos del mundo en constante transición. Su evolución desde una publicación impresa en papel hasta su presencia en los portales más prestigiosos a nivel internacional son reflejo del arduo trabajo y dedicación de todos los involucrados en esta invaluable universidad con más de 400 años de reconocimiento académico, convirtiéndose en un pilar fundamental en la difusión de la investigación y producción de conocimientos de las ciencias de la salud hispanoamericanas, desde el ámbito académico de la Facultad de Ciencias Médicas, Universidad Nacional de Córdoba.

Que estos 80 años sean solo el preludio de un futuro aún más brillante para la revista y su contribución al avance de las ciencias de la salud.

*Las editoras técnicas de la Revista de la Facultad de Ciencias Médicas de Córdoba*

## Notas

[1] Parte de un sistema informático o aplicación que no es visible para el usuario final. Allí se define cómo funciona el sistema, procesamiento y almacenamiento de datos, etc.

[2] Es la parte visible de una aplicación informática, sitio web o software con la que interactúa el usuario final, la interface gráfica

[3] Identificador único y permanente para objetos digitales que facilita la identificación y acceso a nivel mundial.

